# [Retracted] MicroRNA‑365b‑3p represses the proliferation and promotes the apoptosis of non‑small cell lung cancer cells by targeting PPP5C

**DOI:** 10.3892/ol.2023.14010

**Published:** 2023-08-14

**Authors:** Xiaomiao Zhang, Jin Wang, Yuqin Pan, Jun Zhao, Yingge Pan, Yunqi Yan, Zhenya Shen

Oncol Lett 21: 389, 2021; DOI: 10.3892/ol.2021.12650

Following the publication of this paper, we received a request from the authors that the paper be withdrawn on account of the fact that mistakes were made both during the process of specimen collection and in analyzing the data of the article. After having conducted an independent investigation of this paper in the Editorial Office, it was discovered that certain data in Fig. 2 were shared with the following paper, which had already been published by different authors at a different research institute, and which itself has subsequently been retracted: Huang X, Zhou W, Zhang Y and Liu Y: High expression of PTGR1 promotes NSCLC cell growth via positive regulation of cyclin-dependent protein kinase complex. Biomed Res Int: 5230642, 2016.

On account of the fact that certain data in this paper had already been published in another article, and in line with the authors’ request, the Editor of *Oncology Letters* has determined that this paper should be retracted from the journal. The Editor apologizes to the readership for any inconvenience caused.

